# Epilepsy Disclosure Before Marriage: The Collision of Stigma and Ethics

**DOI:** 10.12669/pjms.41.8.12756

**Published:** 2025-08

**Authors:** Wajid Jawaid

**Affiliations:** Dr. Wajid Jawaid, FCPS Neurology Department of Neurology, Dow University of Health Sciences, Karachi, Pakistan

Epilepsy is one of the most common neurological disorders, yet it remains widely misunderstood. In many cultures, especially in South Asia, families struggle with whether to disclose epilepsy before marriage, fearing rejection or social stigma. Although epilepsy affects both men and women, the societal norms in local settings place a much greater burden of expectations of *perfection* on women compared to men.^1^

This dilemma presents a conflict between autonomy and justice- the right to privacy versus the prospective spouse’s right to informed decision-making. While truthfulness aligns with ethical principles, stigma makes disclosure difficult.

The author has seen over a dozen divorces where epilepsy was concealed before marriage and later discovered. At the same time, countless parents are paralyzed by fear, worrying that if they reveal their daughter’s condition, she will never be accepted for marriage. Even more troubling, even parents of infant girls with epilepsy often express anxiety, wondering, “What will happen if she doesn’t recover before she is of marriageable age?” The fact that such fears begin so early highlights how deeply stigma shapes marital and medical decisions.^2^,^3^

Non-disclosure may seem protective, but it may lead to marital distress, legal battles, and deep resentment. Some partners file for divorce, while others struggle to accept a diagnosis they were unprepared for. Families justify concealment by claiming they act “in the daughter’s best interest”, but does a marriage built on secrecy truly serve anyone’s best interest?

This write-up explores the ethical, cultural, and practical aspects of epilepsy disclosure, arguing that honesty is ethically necessary for trust, stability, and informed decision-making.

## THE ETHICAL DILEMMA OF DISCLOSURE

At its core, this debate is about privacy versus ethical responsibility. Individuals have a right to keep medical information private, but a prospective spouse has a right to make an informed marital decision.

Fear of rejection is a primary reason for non-disclosure, yet concealment often leads to a greater crisis later. The ethical question is: *Does avoiding short-term discomfort justify long-term harm?*

## THE ETHICAL PRINCIPLES IN PLAY^4^

### Autonomy: The Right to Make Informed Choices

Every individual has the right to make informed decisions. A spouse should know about a health condition that could impact marital life. Concealing epilepsy violates autonomy, damages trust and often leads to resentment and broken relationships.

### Beneficence: Promoting Well-Being

A marriage is a partnership, and disclosure allows both individuals to prepare for epilepsy’s medical and emotional impact. Unexpected seizures can cause panic and strain relationships. Early disclosure promotes understanding, preparation, and mutual support, strengthening the marriage.

### Non-Maleficence: Preventing Harm

Many choose non-disclosure to avoid harm, but concealment often causes greater harm later. Patients often skip medication to hide epilepsy which worsens their seizures.^2^ If epilepsy is discovered during an emergency, it causes far greater emotional and psychological damage. Ethical transparency prevents this harm.

### Justice: Ensuring Fairness and Social Equity

Concealing epilepsy places an unfair burden on the spouse, who may feel misled or unprepared. Avoiding disclosure reinforces stigma rather than challenging it. Justice extends beyond individual relationships; normalizing epilepsy discussions bring broader cultural change toward fairness and acceptance.

## A PROACTIVE APPROACH: ENCOURAGING ETHICAL DISCLOSURE

The author strongly advocates for full disclosure, but it is also important to recognize that for many families, this is not an easy choice. To support them, following measures may be employed:

- A nationwide campaign by the medical community in general and neurology community in particular to portray epilepsy as a treatable disease rather than a stigma or a taboo.
- Offering free counseling for the potential spouse or in-laws of the families considering disclosure.
- Using social media platforms to spread awareness and combat misinformation.
- Clear communication from the religious clerics that epilepsy is not a disease that may justify rejection of a woman or divorce.
- Examples set by lawmakers and courts in cases that base epilepsy as a reason for divorce.^5^

One message the author always shares with their patients is that if a prospective spouse rejects them after learning about their epilepsy and being counseled, they were never good enough for them to begin with!

Healthcare professionals treating these patients must look at ethical responsibilities, emotional impact and medical considerations. They must guide parents as to when and how to disclose epilepsy while considering marriage proposals for girls, and emphasize that disclosure before marriage establishes trust. Speaking with others who have faced and disclosed similar conditions before marriage may also help ease fears and provide strategies.

## CONCLUSION

The ethics of epilepsy disclosure before marriage are deeply tied to stigma, misinformation, and cultural biases. While concealment may seem protective, it often leads to greater harm in the long run. Transparency strengthens relationships, empowers informed decision-making, and helps dismantle harmful misconceptions. By advocating for honesty, we contribute to a future where epilepsy is treated as a manageable condition rather than a reason for rejection.
